# Nasopharyngeal carriage and antibiotic susceptibility patterns of *streptococcus pneumoniae*, haemophilus influenzae, moraxella catarrhalis and *staphylococcus aureus* among urban Ugandan children post-PCV10 introduction: a cross-sectional study

**DOI:** 10.4314/ahs.v23i4.24

**Published:** 2023-12

**Authors:** Thaddée Nshimiyimana, Christine Florence Najjuka, Winnie Nalwanga, George Katende, David Patrick Kateete

**Affiliations:** 1 Department of Medical Microbiology, Makerere University College of Health Sciences, Kampala, Uganda; 2 Department of Biomedical Laboratory Sciences, School of Health Sciences, College of Medicine and Health Sciences, University of Rwanda, Kigali; 3 Department of Immunology and Molecular Biology, Makerere University College of Health Sciences, Kampala, Uganda

**Keywords:** Antibiotic resistance, Bacterial colonization, Bacterial isolates

## Abstract

In 2013, Uganda introduced the PCV10 pneumococcal vaccine and it is given to children at 6, 10 and 14 weeks after birth. Carriage prevalence studies post PCV10-introduction are necessary for monitoring the impact of vaccination and trends in antibiotic resistance. Here, we studied carriage/antibiotic resistance of Streptococcus pneumoniae (pneumococcus), *Haemophilus influenzae, Moraxella catarrhalis*, and *Staphylococcus aureus* isolated from 194 children at the Mulago Assessment Centre clinic in Kampala-Uganda, 5 years post-PCV10 introduction. Almost all the children were vaccinated with PCV10 (98.5%, 191/194). The overall carriage prevalence (any species) was 62% (120/194), and it was associated with a history of antibiotics use (p=0.0159) and having respiratory symptoms (p=0.0003). The pneumococcus, *H. influenzae, M. catarrhalis*, and *S. aureus* carriage prevalence was 46% (90/194), 21% (40/194), 7% (14/194), and 6% (12/194), respectively. Species co-carriage occurred in 32 children (17%, 32/194), predominantly multidrug resistant pneumococcus + *H. influenzae* (23 children). Furthermore, pneumococci were highly resistant to cotrimoxazole (100%), erythromycin (76%), and tetracycline (52%), 42% being multidrug-resistant. Overall, we note an increase in antibiotic resistance post-PCV10 introduction, and microbial shifts i.e., a decrease in pneumococcus, *M. catarrhalis* and *S. aureus* carriage and an increase in *H. influenzae* carriage suggesting vaccine-associated perturbation of the respiratory ecology.

## Introduction

The human nasopharynx harbors potentially pathogenic bacteria like Streptococcus pneumoniae (pneumococcus), *Haemophilus influenzae, Moraxella catarrhalis* and *Staphylococcus aureus*,^53^ which can migrate to other body parts and cause invasive disease e.g., pneumonia and meningitis, or localized infection e.g., otitis media and sinusitis. Invasive pneumococcal disease (IPD) and *H. influenzae* meningitis are major causes of death in young children globally.^15^,^17^ *S. aureus* is commonly associated with pneumonia, endocarditis, osteomyelitis, skin and bloodstream infections^49^,^52^ while *M. catarrhalis* is associated with sinusitis and acute exacerbations of chronic obstructive pulmonary disease.^38^

Nasopharyngeal colonization by bacteria like the pneumococcus, *H. influenzae, M. catarrhalis* and *S. aureus* precedes invasive disease and generally infection,^25^ and it is linked to several factors such as crowding, upper respiratory tract infections, frequent antibiotics use, immunization, etc.^17^,^32^,^41^,^49^ Importantly, IPD and *H. influenzae* meningitis are associated with a few well-known serotypes/strains and vaccination, for example with pneumococcal conjugate vaccines (PCVs) and/or *H. influenzae* type b (Hib) vaccine, has reduced the disease incidence and frequency of carriage of vaccine-type (VT) serotypes/strains in populations.^18^,^19^,^36^,^55^ However, while vaccination has reduced the global pneumococcal- and *H. influenzae*-related deaths (for example to approx. 51% and 90% respectively, between 2000 to 2015),^54^ the death toll attributed to infection with these organisms remains high e.g., in 2015, 294,000 and 29,500 pneumococcal- and *H. influenzae*-related deaths respectively, were reported in children.^54^

Additionally, all the aforementioned bacteria have complex antibiotic resistance mechanisms that threaten treatment of infection for example, pneumococci are associated with reduced susceptibility to penicillin, chloramphenicol, and trimethoprim-sulfamethoxazole. Moreover, the introduction of PCVs (i.e., PCV7, PCV10, and PCV13) has been associated with replacement of VT serotypes with non-vaccine-type (NVT) serotypes and disruption of the respiratory tract ecology, which affects microbial interactions within a niche.^50^,^52^ Case in point is the well-documented antagonism in coexistence between *S. pneumoniae* and *S. aureus* in PCV-vaccinated healthy children;^6^,^36^ interestingly, this inverse relationship is abolished in children living with HIV i.e., *S. pneumoniae* and *S. aureus* coexist,^50^ probably due to a comprised immune system response associated with HIV infection. In Africa where the HIV burden is high, vaccine-associated perturbations could predispose children to higher *S. aureus* colonization, which is associated with staphylococcal infections including infection with drug resistant strains like methicillin resistant *S. aureus* (MRSA).

IPD is a frequent cause of death in children in Uganda.^29^ In 2013, PCV10 was formally introduced into the country's routine immunization schedule to avert the infant and child deaths due to pneumococcal disease.^39^,^48^ The vaccine is administered to children less than 1 year of age at 6, 10 and 14 weeks. With time, the Uganda's Ministry of Health expects PCV-vaccination to prevent ∼840 and ∼94,071 annual childhood cases attributed to pneumococcal meningitis and pneumonia respectively, and save ∼465 and ∼10,796 lives that could have been lost.^1^ Information on pneumococcal carriage post-PCV introduction is essential – not only in monitoring the impact of vaccines, but also for insights into antimicrobial resistance and respiratory tract microbial shifts following vaccination. Therefore, this study aimed to determine the pneumococcal carriage, species co-carriage and antibiotic resistance patterns of potential respiratory bacterial pathogens isolated from urban Ugandan children less than 5 years of age post-PCV10 introduction.

## Materials and methods

### Study setting and participants

This cross-sectional study was conducted at the Mulago Hospital Assessment Centre clinic^21^ in Kampala Uganda, between March and May, 2019. The Assessment Centre is the first contact clinic for referred and non-referred patients visiting the hospital^17^ and it receives a large number of patients including outpatient children. The study participants were children 6-60 months of age who attended the children's outpatient clinic at the Assessment Centre. We estimated the sample size using formulae for prevalence studies;^40^ a pneumococcal carriage prevalence of 20% (according to previous studies on pneumococcal carriage in Uganda^46^) was used to estimate the sample size of 246 children. Systematic random sampling with replacement was used to select the 246 children. A trained nurse recruited the children; parents/guardians of the recruited children were assented and an interviewer-administered questionnaire was used to collect clinical and demographic data. Almost all the children were vaccinated with PCV10 i.e., 99% (243/246).

### Sample collection, culturing and species identification

Nasopharyngeal samples were collected from the children by using single sterile swabs (1 SP7D - Dacron Medical Packaging Swab-Pak^™^, USA) according to the World Health Organization (WHO) guidelines^47^ Briefly, the child's head was slowly tilted backwards and the swab passed directly backwards – parallel to the base of the nasopharynx passage. Once in the nasopharynx, the swab was rotated at 180° to be saturated and removed slowly, and immediately inserted into a tube with 1 ml of STGG medium (i.e., 2g/100 ml Skim milk powder, 3g/100 ml Tryptone soya broth, 0.5% Glucose, & 85% Glycerol), labeled unambiguously and sent to the Clinical Microbiology Laboratory at Makerere University College of Health Sciences located on the same campus as the hospital. The Clinical Microbiology Laboratory is certified by the College of American Pathologists (CAP) and performs standard microbiological culturing and antimicrobial susceptibility testing. Within 3 hours, the nasopharyngeal-STGG samples were processed for culturing i.e., vortex briefly (∼10s), streak on 5% sheep blood agar and/or chocolate agar media and incubate at 37°C under 5-10% CO^2^ for 24-48 hours. Respiratory bacteria were detected according to standard microbiology methods with focus on the most common potential pathogens i.e., *S. pneumoniae, H. influenzae, M. catarrhalis*, and *S. aureus*. Only one colony per plate was analysed except where distinct morphological differences were noted.

Briefly, *S. pneumoniae* was identified according to the WHO guidelines^47^,^56^ i.e., colony characteristics (small, grey, alpha-hemolytic glistering colonies), Gram staining properties (i.e., positive diplococci) and biochemical characteristics (bile solubility and catalase reaction). Also, an optochin sensitivity test i.e., a disc impregnated with optochin, placed in the first streak area and the plate incubated for 24-48 hours at 37°C under 5%-10% CO2, was done; presence of an inhibition zone greater than 14 mm around the optochin disc allowed us to presumptively identify *S. pneumoniae*. For inhibition less than 14 mm, we performed the bile solubility test; if the turbid suspension is cleared in 2% sodium deoxycholate after 10-15 min incubation at 35-37°C, the isolate was identified as S. *pneumoniae*. For *H. influenzae*, we followed approaches commonly used in our setting^43^ i.e., identify presumptive *H. influenzae* isolates on chocolate agar medium according to colony characteristics (small, smooth, convex, pale, grey, or transparent colonies), oxidase test, Gram staining properties (pleomorphic Gram negative rods or coccobacili), and growth dependence on both hemin (X factor) and nicotinamide adenine dinucleotide (NAD) on Mueller-Hinton agar (MHA).^56^ Likewise, *M. catarrhalis* was identified based on Gram staining properties and colony characteristics i.e., large, non-pigmented or grey, opaque colonies, occasionally smooth with a friable hockey puck consistency when pushed over the surface of the agar medium.^35^ As well, the oxidase test, catalase test, DNAse test, growth at room temperature on nutrient agar, and butyrate esterase enzyme production were performed to confirm isolates as *M. catarrhalis*. Finally, *S. aureus* identified as previously described, using a tri-combination method of subjecting presumptive isolates to growth on Mannitol Salt Agar and DNAse agar, and tube coagulase testing.^26^ Following confirmation of the bacterial species of interest, isolates were stored by cryopreservation at -80 ^0^C prior to further analyses. However, upon retrieval of isolates for analysis, 52 isolates from 52 children were not recovered leaving us with 194 isolates from the same number of children.

### Antibiotic susceptibility testing

Bacterial colonies from a 24-hour old subculture were suspended in sterile normal saline to achieve a turbidity equivalent to 0.5 McFarland standard, and prepared for drug sensitivity testing using the disc diffusion method on MHA medium supplemented with 5% sheep blood. Following inoculation on MHA, antibiotic discs and/or E-test strips were placed on the streaked MHA, and plates incubated for 24 hours at 35-37°C under 5%-10% CO_2_. Inhibition zone diameters and minimum inhibitory concentrations (MICs) for various antibiotics were read and interpreted according to the Clinical and Laboratory Standards Institute (CLSI) guidelines and Bell et al recommendation for M. catarrhalis^4^,^9^ (summarized in Table S1). Antibiotic discs and E-test strips were purchased from Oxoid limited (UK).

*S. pneumoniae* isolates were tested for susceptibility to oxacillin (1µg), erythromycin (15µg), clindamycin (2µg), tetracycline (30µg), chloramphenicol (30µg) and trimethoprim/sulfamethoxazole (co-trimoxazole) (1.25µg/23.5µg). Additionally, E-tests for penicillin G (P 32 µg/ml-0.002 µg/ml) and ceftriaxone (CRO 32µg/ml-0.002µg/ml) were performed on isolates with ≤19 mm of inhibition zone diameter around the oxacillin disc. *H. influenzae* and was tested for susceptibility to ampicillin (10µg), Augmentin (30µg), tetracycline (30µg), chloramphenicol (30µg), co-trimoxazole (1.25/23.5µg), ciprofloxacin (5µg), cefuroxime (30µg) and ceftriaxone (30µg), so are S. aureus and *M. catarrhalis* susceptibility to penicillin G (10 units), cefoxitin (30µg), erythromycin (15µg), co-trimoxazole (1.25/23.75µg), tetracycline (30µg), ciprofloxacin (5µg), chloramphenicol (50µg), gentamicin (10µg) and clindamycin (2µg). To quality-control the assays, reference strains ATCC49619 (*S. pneumoniae*), ATCC49247 (*H. influenzae*) and ATCC25923 (*S. aureus*) were used.

### Statistical analysis

All data were entered into a data capture tool Epidata (epidataentryclient.4.4.3.1.win.64) and exported to SPSS v17.0 for analysis. We used descriptive statistics to analyse categorical data summarized as proportions and presented as percentages. Quantitative data were summarized as mean/median, mode, minimum and maximum. Chi-square test was used to determine whether there is a statistically significant difference between the expected frequencies and the observed frequencies in categories of a contingency table. For all comparisons, a p-value of ≤0.05 at multivariate level was considered significant.

### Ethical approvals

This study was approved by the Makerere University School of Biomedical Sciences Research and Ethics Committee (Approval # SBS-620), and the Mulago National Referral Hospital Research and Ethics Committee (Approval # MHREC1574). The parents of the children provided written informed assent.

## Results

### Demographics

The demographic characteristics of the children are listed in Table 1. Briefly, female and male children were 46.4% (90/194) and 53.6% (104/194), respectively; almost all (98.5%, 191/194) the children were vaccinated with PCV10 and residing in greater Kampala metropolitan area (i.e., Kampala city proper and neighbouring suburbs in the districts of Wakiso, Mukono, Mpigi, Buikwe and Luweero). The youngest child was 6 months old while the oldest was 60 months (i.e., 5 years old); the median and modal age were 21 and 36 months, respectively. According to information from parents/caretakers, the reasons children sought care were fever (53%, 102/194), respiratory symptoms (27%, 52/194), and diarrhoea (21%, 40/194); however, we did not associate bacterial isolates or carriage rates with disease as this was beyond the scope of the study.

**Table 1:** Demographic characteristics of the children (N=194) and factors associated with bacterial carriage

Characteristic, n (%)	No carriage, n=74 (%)	With carriage, n=120 (%)	*X*^2^ (1, N=194), (p-value*)
*Gender*			
Male, 104 (54)	34/104 (33)	70/104 (67)	2.82, (0.092852)
Female, 90 (46)	40/90 (44)	50/90 (56)	
*Age in months*			
=10, 44 (23)	15/44 (34)	29/44 (66)	0.80 (0.848785)
11-20, 69 (36)	26/69 (38)	43/69 (62)	
21-30, 39 (20)	17/39 (44)	22/39 (56)	
31-60, 42 (22)	16/42 (38)	26/42 (62)	
*Overweight*			
Yes, 19 (10)	5/19 (26)	14/19 (74)	1.24 (0.263739)
No, 175 (90)	69/175 (39)	106/175 (61)	
*Underweight*			
Yes, 17 (9)	9/17 (53)	8/17 (47)	1.72 (0.188533)
No, 177 (91)	65/177 (37)	112/177 (63)	
*Stunting*			
Yes, 59 (30)	19/59 (32)	40/59 (68)	1.26 (0.260086)
No, 135 (70)	55/135 (41)	80/135 (59)	
*Wasting*			
Yes, 11 (6)	5/11 (46)	6/11 (55)	0.26 (0.607307)
No, 183 (94)	69/183 (38)	114/183 (62)	
*No. of persons in household*			
=4, 122 (63)	45/122 (37)	77/122 (63)	0.22 (0.638381)
5-8, 72 (37)	29/72 (40)	43/72 (60)	
*School attendance*			
Yes, 123 (63)	48/123 (39)	75/123 (61)	0.11 (0.739777)
No, 71 (37)	26/71 (37)	45/71 (63)	
*History of antibiotics use*			
Yes, 108 (56)	33/108 (31)	75/108 (69)	5.95 **(0.014747)**
No, 86 (44)	41/86 (48)	45/86 (52)	
*Allergy*			
Yes, 43 (22)	17/43 (40)	26/43 (61)	0.04 (0.831499)
No, 151 (78)	57/151 (38)	94/151 (62)	
HIV exposure**			
Yes, 7 (4)	5/7 (71)	2/7 (29)	3.40 (0.064811)
No, 187 (96)	69/187 (37)	118/187 (63)	
Respiratory symptoms			
Yes, 141 (73)	43 (31)	98/141 (70)	12.79 **(0.000348)**
No, 53 (23)	31 (59)	22/53 (42)	
*Previous hospitalization*			
Yes, 48 (25)	21/48 (44)	27/48 (56)	0.84 (0.356709)
No, 146 (75)	53/146 (36)	93/146 (64)	
*Smoking exposure*			
Yes, 37 (19)	14/37 (38)	23/37 (62.2)	0.0018 (0.965969.)
No, 157 (81)	60/157 (38)	97/157 (62)	

X^2^ = Chi square *p-values depict differences with respect to carriage-status only **Refers to mothers living with HIV but not sure about the HIV status of their children

### Carriage prevalence of common respiratory bacteria and factors associated with carriage

The overall prevalence of carriage (i.e., any species detected) was 62% (120/194) and the difference was not statistically significant regarding gender. Overall, bacterial carriage was associated with a history of antibiotics use (p=0.0159) and having respiratory symptoms (p=0.0003), Table 1; several other risk factors for carriage were assessed and found not to be statistically significant, Table 2. Regarding individual species, the carriage prevalence was 46% (90/194), 21% (40/194), 7% (14/194), and 6% (12/194) for *S. pneumoniae, H. influenzae, M. catarrhalis*, and *S. aureus*, respectively. A single species was identified in 88 children (45%, 88/194), two species in 28 children (14%, 28/194), and three species in four children (2%, 4/194). Overall, species co-carriage (i.e., carriage of two or more species) was noted in 32 children (17%, 32/194) with *S. pneumoniae* + *H. influenzae* as the most common co-carriage pattern (23 children), Table 2. Interestingly, *S. pneumoniae* + *S. aureus* co-carriage was not detected. Further, 21 of the co-colonized children carried multidrug resistant bacteria mainly *S. pneumoniae* and *H. influenzae*.

**Table 2:** Carriage prevalence of *S. pneumoniae, H. influenzae, M. catarrhalis* and *S. aureus* among urban Ugandan children post-PCV10 introduction (n=194)

Species	Frequency	Percent
Total carriage for all bacterial species of interest	120	62
Pneumococcus overall carriage	90	46
*H. influenzae* overall carriage	40	21
*M. catarrhalis* overall carriage	14	7
*S. aureus* overall carriage	12	6
Colonized by *S. pneumoniae* only	61	31
Colonized by *H. influenzae* only	11	6
Colonized by *S. aureus* only	8	4
Colonized by *M. catarrhalis* only	8	4
Colonized by *S. pneumoniae* + *H. influenzae*	23	12
Colonized by *S. pneumoniae* + *M. catarrhalis*	2	1
Colonize by *H. influenzae* + *M. catarrhalis*	2	1
Colonized by *H. influenzae + S. aureus*	1	0.5
Colonized by *S. pneumoniae* + *S. aureus*	0	0
*S. pneumoniae + H. influenzae* + *S. aureus*	2	1
*S. pneumoniae + H. influenzae + M. catarrhalis*	1	0.5
*S. pneumoniae + S. aureus + M. catarrhalis*	1	0.5
Overall carriage of a single species	88	45
Overall co-carriage of 2 species	28	14
Overall co-carriage of 3 species	4	2
Overall co-carriage of 2 or 3 species	32	17
No carriage	74	38

### Antibiotic susceptibility profiles

The antibiotic susceptibility patterns are shown in Table 3. Briefly, all pneumococci (100%, 90/90) were non-susceptible to penicillin at ≥0.06 mg/l MIC but susceptible at ≤2 mg/l MIC with the exception of one isolate that had intermediate resistance (1%, 1/90), Table 3. Similarly, at ≤1 mg/l MIC for ceftriaxone, pneumococci (99%, 89/90) were susceptible except one isolate that had intermediate resistance (1%, 1/90); at ≤0.5 mg/l MIC, 82 isolates (91%, 82/90) were susceptible, seven (8%, 7/90) had intermediate resistance while one was resistant, Table 3. The MIC50 and MIC90 for penicillin were 0.5 mg/l and 1 mg/l, respectively; for ceftriaxone, MIC50 and MIC90 were 0.25 mg/l and 0.56 mg/l, respectively. Overall, pneumococci were highly resistant to co-trimoxazole (100%), erythromycin (76%), and tetracycline (52%), and 42% (81/194) were multidrug resistant (resistance to three or more classes of antimicrobials).

**Table 3:** Antibiotic susceptibility patterns of the bacteria isolated from the children (n=194)

Antibiotic	*S. pneumoniae*, n=90 (%)			*H. influenzae*, n=40 (%)			*M. catarrhalis*, n=14 (%)			*S. aureus*, n=12 (%)		
	S	I	R	S	I	R	S	I	R	S	I	R
Chloramphenicol	66 (73)	1 (1)	23 (26)	21 (53)	6 (15)	13 (33)	14 (100)	0	0	12 (100)	0	0
Tetracycline	32 (35)	13 (14)	**47 (52)**	1 (3)	9 (23)	**30 (75)**	9 (64)	4 (29)	1 (7)	9 (75)	0	3 (25)
SXT	0	0	**90 (100)**	0	0	40 **(100)**	0	0	14 **(100)**	0	1 (8)	**11 (92)**
Clindamycin	63 (70)	6 (7)	23 (26)	-	-	-	-	-	-	5 (42)	0	7 **(58)**
Erythromycin	18 (20)	6 (7)	**68 (76)**	-	-	-	-	-	-	3 (25)	0	**9 (75)**
Penicillin (≤2mg/l)	89 (99)	1 (1)	0	-	-	-	0	0	**14 (100)**	0	0	**12 (100)**
Penicillin (≥0.06 mg/l)*	0	0	**90 (100)**	-			-	-	-	-	-	-
Ceftriaxone (≤1mg/l)	89 (99)	1 (1)	0	26 (65)	0	14 (35)	14 (100)	0	0	-	-	-
Ceftriaxone (≤0.5 mg/l) *	82 (91)	7 (8)	1 (1)				-	-	-	-	-	-
Ampicillin	-	-	-	17 (43)	4 (10)	**19 (48)**	0	0	**14 (100)**	-	-	-
Ciprofloxacin	-	-	-	38 (95)	-	2 (5)	14 (100)	0	0	8 (67)	-	4 (33)
Gentamicin	-	-	-	-	-	-	14 (100)	0	0	9 (75)	1 (8)	2 (17)
AMC	-	-	-	27 (68.6)	0	13 (33)	14 (100)	0	0	-	-	-
Cefuroxime	-	-	-	30 (75)	2 (5)	8 (20)	14 (100)	0	0	-	-	-
MDR	**50 (56)**			**22 (55)**			0			**9 (75)**		

S, Susceptible; I, Intermediate; R, Resistant SXT, trimethoprim-sulfamethoxazole; AMC, amoxicillin-clavulanate (Augmentin); MDR, multidrug resistant * CLSI interpretation guidelines for *S. pneumoniae* by minimum inhibitory concentration (MIC) susceptibility testing method - Not applicable

Additionally, we found H. influenzae isolates to be highly resistant to co-trimoxazole (100%, 40/40) and tetracycline (75%, 30/40) but generally susceptible to ciprofloxacin and cefuroxime, Table 3. Multidrug resistance among *H. influenzae* was noted in 22 isolates (55%, 22/40). Further, with the exception of ampicillin, penicillin and co-trimoxazole to which resistance was 100%, nearly all *M. catarrhalis* isolates were susceptible to the antibiotics tested, Table 3. For *S. aureus*, all isolates were susceptible to chloramphenicol and cefoxitin/oxacillin thus, MRSA was not detected. However, *S. aureus* was resistant to penicillin (100%) and co-trimoxazole (with the exception of one isolate for the latter); likewise, resistance to erythromycin, clindamycin, and penicillin among *S. aureus* was high, Table 3. Inducible clindamycin resistance was noted in 42% (5/12) of the S. aureus isolates.

Of note, a comparison of S. *pneumoniae, H. influenzae, M. catarrhalis* and *S. aureus* carriage before and after introduction of PCV10 reveals a reduction in pneumococcal and *H. influenzae* carriage prevalence post-PCV10 introduction while *M. catarrhalis* carriage prevalence increased, Table 4. Furthermore, there was an increase in resistance to co-trimoxazole, tetracycline, chloramphenicol, erythromycin and clindamycin post-PCV10 introduction compared to the pre-PCV era (i.e., 199520 when pneumococcal vaccination was non-existent in Uganda), Table 5.

**Table 4:** A comparison of nasopharyngeal carriage of *S. pneumoniae, H. influenzae, M. catarrhalis* and *S. aureus* by Ugandan children under 5 years of age before and after introduction of PCV10

Bacteria identified	Pre-PCV10, n (%)	Post-PCV10, n (%)	*X*^2^(1, N=194), (p-value*)
	*Joloba et al*^20^, n=191	This study, n=194	
Total carriage for bacterial species of interest	-	120 (62)	
*S. pneumoniae*	118 (62)	90 (46)	9.179 (**0.002452**)
*H. influenzae*	-	40 (21)	
*M. catarrhalis*	-	14 (7)	
*S. aureus*	-	12 (6)	
	*Rutebemberwa et at*^46^, n=152		
Total carriage for bacterial species of interest	100 (66)	120 (62)	0.56 (0.450426)
*S. pneumoniae*	89 (59)	90 (46)	5.04 (**0.024661**)
*H. influenzae*	16 (11)	40 (21)	6.39 (**0.011419**)
*M. catarrhalis*	23 (15)	14 (7)	119.19 (**<<0.00001**)
*S. aureus*	-	12 (6)	
*Carriage of 2 species*	30 (20)	28 (14)	1.7182 (0.18992)
*Carriage of 3 species*	0	4 (2)	
*No carriage*	52 (34)	74 (38)	
	*Lindstrand et al*^33^, n=1723		
Total carriage of all bacteria	-	120 (62)	
*S. pneumoniae*	957 (56)	90 (46)	5.89 (**0.015221**)
*H. influenzae*	-	40 (21)	
*M. catarrhalis*	-	14 (7)	
*S. aureus*	-	12 (6)	
	*Nackers et al*^39^, n=387		
Total carriage of all bacteria	-	120 (62)	
*S. pneumoniae*	298 (77)	90 (46)	54.58 (**<0.00001**)
*H. influenzae*	-	40 (21)	
*M. catarrhalis*	-	14 (7)	
*S. aureus*	-	12 (6)	
		Omoding et al^43^, n=248	
Total carriage of all bacteria			
*S. pneumoniae*	NA	-	
*H. influenzae*		51 (21)	40 (21) (=1.0000)
*M. catarrhalis*	NA	-	
*S. aureus*	NA	-	

NA, not applicable; -, not reported by the investigators

**Table 5:** A comparison of antibiotic susceptibility profiles of *S. pneumoniae* in 1995 (prior to introduction of PCV-10) and 2019 (post-PCV10 introduction)

Antibiotic	Joloba et al (1995)^20^, n=115 (%)			This study (2019), n=90 (%)		
	S	I	R	S	I	R
Penicillin	19 (17)	96 (84)	0	89 (99)	1 (1)	0
SXT	17 (15)	2 (2)	96 **(84)**	0	0	90 **(100)**
Tetracycline	78 (68)	4 (4)	33 (29)	32 (35)	13 (14)	**47 (52)**
Chloramphenicol	103 (87)	0	12 (11)	66 (73)	1 (1)	23 **(26)**
Erythromycin	115 (100)	0	0	18 (20)	6 (7)	**68 (76)**
Clindamycin	115 (100)	0	0	63 (70)	6 (7)	23 **(26)**

S, Susceptible; I, Intermediate; R, Resistant SXT, trimethoprim-sulfamethoxazole

## Discussion

In this study, the overall bacterial carriage prevalence was high at 62%, and we found a significant association between bacterial carriage and a history of antibiotics use and having respiratory symptoms. Although there are limited studies in Africa on carriage prevalence of the aforementioned bacteria, higher bacterial carriage rates have been reported in the western hemisphere,^31^ and the difference might be attributed to regional variations. Overall, we found the pneumococcal carriage prevalence (i.e., 46% [90/194]) in vaccinated urban Ugandan children to be low compared to the pre-PCV era.^20^,^33^,^39^,^46^ The pooled pneumococcal carriage prevalence pre-PCV10 introduction is 64.8% and 47.8% for low-income countries and lower middle-income countries respectively,^2^ which is comparable to the reported prevalence before PCV10 rollout in Uganda i.e., 62% (118/191) in 2001;^20^ 58.6% (89/152) in 2008^46^; 56% (957/1723) in 2008/2009/2011;^33^ and 77% (297/387) in 2014.^39^ Although we found a decrease in pneumococcal carriage, it is well documented that vaccination with PCVs only leads to a decrease in carriage of VT serotypes but has no effect on the overall pneumococcal carriage^27^,^45^,^50^ in that VT serotypes are replaced with non-VT serotypes following vaccination.^27^,^45^,^50^ Intriguingly, the serotype coverage for PCV10 among Ugandan children was found to be low (i.e., 33%39 to 42%33), which may affect the vaccine's effectiveness in protecting children against IPD. Indeed, there are emerging reports of high carriage of VT sero-types years post-PCV introduction in Africa compared to high-income settings,^51^ implying that PCVs could be sub-optimally effective at preventing IPD in low-income countries. For example, in the Dominican Republic, the carriage prevalence of VT serotypes in children was higher (60%) than the pre-vaccine carriage prevalence (40%) 3 years post-PCV13 introduction^12^.

Furthermore, the *H. influenzae* carriage prevalence (21%) reported in this study is similar to findings by Omoding et al who reported 20.56% H. influenzae carriage in children under 5 years in Mbarara city, south-western Uganda.^43^ Additionally, species co-carriage, mainly multidrug resistant *S. pneumoniae* + *H. influenzae*, was noted in the vaccinated children. While detection of co-carriage alludes to the existence of interactions among the respiratory bacteria,^7^,^11^ vaccination with PCVs is associated with perturbations in the upper respiratory tract ecology^7^,^11^ and flora replacement, including replacement with abnormal/drug resistant bacteria. Generally, the species co-carriage patterns observed in this study were described before for children in rural eastern Uganda before PCV10 introduction,^46^ except that S. aureus and co-carriage of three species simultaneously was not reported.^46^ Apparently, the species co-carriage prevalence (≤17%) in Ugandan children is somewhat low compared to other countries i.e., 60% (97/161) co-carriage in Fiji where colonization by a single species was 27% (44/161)^13^; 25.9%-54.1% and 27.4%-57.8% co-carriage in France for *H. influenzae* + *S. pneumoniae* respectively, pre- and post-PCV introduction;^3^,^10^ 33% in Hungary^31^; and 29% and 16.1% co-carriage in Japan for *S. pneumoniae + H. influenzae and S. pneumoniae + H. influenzae + M. catarrhalis*, respectively.^42^ The difference in carriage could be attributed to regional variation in bacterial carriage, seasonal variations, pneumococcal vaccination or antibiotic exposure.^14^,^16^ Generally, our data show microbial shifts in urban sick Ugandan children 5 years post-PCV10 vaccination, with a decrease in *S. pneumoniae, M. catarrhalis* and *S. aureus* carriage and an increase in *H. influenzae* carriage. In contrast, in Fiji, vaccination of young children with PCV7 did not affect nasopharyngeal carriage of *S. pneumoniae, H. influenzae, M. catarrhalis*, and *S. aureus*.^13^

Furthermore, *H. influenzae* isolates in this study were highly susceptible to ciprofloxacin and cefuroxime and this been reported before in Uganda.^43^ However, according to other previous studies^20^,^46^ in Uganda there is a general increase in carriage prevalence of drug resistant bacteria post-PCV10 introduction (see Table 4), which could be attributed to ecological disruption associated with PCV vaccination.^7^,^11^ However, the previous Ugandan studies focused on samples from healthy children unlike the current study that studied isolates from sick children hence, caution is necessary while comparing isolates from these studies.

Similar to our findings, a previous study in Uganda reported high-level penicillin resistance among pneumococci (i.e. 83.5%).^20^ Pneumococcal resistance to penicillin is a global concern as penicillin non-susceptible pneumococci are on the WHO's list of bacteria for which there is an urgent need of new therapeutics.^44^ Note that breakpoints for penicillin susceptibility change over time due to clinical outcomes between susceptible and non-susceptible strains, as well as pharmacokinetic/pharmacodynamic properties of the drug and site of infection.^8^,^28^ For instance, based on CLSI's guidelines for meningitis,^9^ our findings reveal high-level penicillin resistance (100%); however, for infection in other body parts, the same susceptibility results are interpreted as 99% susceptible and 1% intermediate resistance. Overall, the non-susceptibility to penicillin by pneumococci in our study contrasts with findings from DR Congo (80%),^5^ Tanzania (67.8%),^37^ and Kenya (81.9%).^30^ For ceftriaxone, similar interpretation guidelines as in meningitis suggest that 8% of the isolates in this study were intermediate while 1% were resistant, in total yielding 9% ceftriaxone non-susceptible isolates. These rates are low compared to reports from DR Congo^5^ but high compared to rates from Kenya^30^ and Tanzania,^37^ where ceftriaxone resistance was not detected. The high co-trimoxazole resistance in this study, similar to a recent study in Uganda,^46^ and other countries in Africa, could be attributed to its wide use as a prophylactic drug in children living with HIV. Furthermore, nearly all *M. catarrhalis* isolates were susceptible to antibiotics with the exception of penicillin, ampicillin and co-trimoxazole to which resistance was 100%; these patterns were reported previously and justified by *M. catarrhalis* capacity to produce β-lactamases.^35^ Overall, our findings reveal an increase in resistance to erythromycin, clindamycin, tetracycline and chloramphenicol in pneumococci post-PCV10 introduction in Uganda,^20^,^46^ and the factors underlying this trend require further study

Co-carriage of the pneumococcus and *S. aureus* was not seen in this study, and *S. aureus* carriage prevalence 5 years post-PCV10 vaccination in the children was unusually low compared to the carriage prevalence pre-PCV10 introduction.^22^-^24^ Though, this finding is in agreement with the well-documented inverse relationship in coexistence between the pneumococcus and *S. aureus* in healthy children without HIV infection post-PCV vaccination.^7^,^34^ All the children in this study were HIV-negative hence, absence of pneumococcus + *S. aureus* co-carriage confirms that this inverse association exclusively occurs in HIV-uninfected children.^7^,^34^ The exact mechanism of the negative relationship is not clear but the contributing factors include inhibition of *S. aureus* by H_2_O_2_ produced by pneumococci, as well as host immunity.^7^,^11^,^34^ Interestingly, we did not expect an inverse association between pneumococci and *S. aureus* as we studied isolates from sick children and this requires further study.

This study had certain limitations. First, 52 isolates were not recovered leaving us with a small sample size of 194 children; key factors that could have led to loss of isolates include (among others), i) power fluctuations that could have led to freeze-thaw episodes, eventually affecting the ability of the cryoprotectant to preserve the isolates and ii) the fastidious nature of the bacterial species investigated, notably *S. pneumoniae* and *H. influenzae*. Nevertheless, several studies have used similar or less sample sizes and obtained meaningful results.^12^,^13^,^27^ Second, we did not serotype pneumococci for a picture of circulating VT serotypes in the children hence, *S. pneumoniae* serotypes in this manuscript are mentioned only in context of i) the fact very few serotypes/strains (of several hundred known so far) are associated with IPD, and ii) vaccination with PCVs leads to replacement of VT serotypes with NVT serotypes. Though, pneumococcal serotypes for Ugandan children pre-PCV10 introduction have been described^33^,^39^ in which a low serotype coverage was reported. Given the findings from Malawi^51^ and Fiji,^13^ we expect VT serotypes in vaccinated Ugandan children 5 years post-PCV10 introduction to be similar to the pre-PCV10 serotypes reported by Nackers et al^39^ and Lindstrand et al^33^. Lastly, although we isolated the investigated bacteria from sick children, we did not perform clinical evaluation to determine whether the isolates were clinically relevant.

## Conclusions

There is a decrease in the overall pneumococcal carriage, and microbial shifts – in this case, a decrease in S. pneumoniae, M. catarrhalis and S. aureus carriage prevalence and an increase in H. influenzae carriage prevalence among vaccinated urban Ugandan children 5 years post-PCV10 introduction. This could be attributed to the effect of PCV introduction on the respiratory tract microbiota and potentially, could impact on the incidence of IPD and antibiotic resistance emergence post-PCV introduction.
